# The Role of Microcirculation in Haemodynamics: A Journey from Atlas to Sisyphus

**DOI:** 10.2478/jccm-2024-0021

**Published:** 2024-04-30

**Authors:** Constantin Bodolea

**Affiliations:** University of Medicine and Pharmacy Iuliu Hatieganu Cluj Napoca, Romania

Microcirculation, through its complex network of vessels (arterioles, capillaries and venules), plays a pivotal role in maintaining cellular functions. Located between the macrocirculatory system- the heart and large vessels, and the interstitial environment, microcirculation acts as a gateway for oxygen supply (O2), removal of carbon dioxide (CO2), delivery of nutrients and hormones, and plays a crucial role in the immune response, thermoregulation and coagulation control.

In order to maintain an adequate supply of oxygen and nutrients to cells and tissues at the levels of the macro-, regional- and microcirculatory fields, the physiological mechanisms that regulate blood perfusion are closely interrelated.

This functional interdependence between macro-haemodynamics, microhaemodynamics, interstitial space and subcellular structures (such as the mitochondria, the bioenergetic factory of the organism) was demonstrated by Dr. Henry Weil, who explicitly defined the condition of acute circulatory failure or circulatory shock [1,2]. In his work, he highlights the catastrophic consequences of arterial hypotension associated with tissue hypoperfusion in various states of shock. This is illustrated by a decrease in the cellular supply of oxygen and by an impaired oxidative phosphorylation. In addition to the etiological classification of shock states, Dr. Weil described microcirculation's behaviour in different types of shock, the haemodynamic response to different fluid therapy strategies, and the utility of invasive haemodynamic monitoring. In other words, it has been identified a dependent link between macrohaemodynamics, microhaemodynamics and the mitochondrial bioenergetic performance. Furthermore, regardless of the source of the primary impairment of haemodynamics (macro- or micro-haemodynamics), the catastrophic consequences will always be directed against an optimal cellular function.

Conventionally, haemodynamic resuscitation is based on restoring macrohaemodynamics by reaching a target blood pressure and cardiac output. However, from a clinical perspective, it is much more challenging to observe changes in the microcirculation during therapeutic optimisation in different types of shock, particularly since microcirculation optimisation and restoration of tissue homeostasis are not always achieved after the normalisation of the macrohaemo-dynamics-phenomenon also known as “the absence of haemodynamic coherence”.

Consequently, haemodynamic coherence between the macro- and microvascular systems, following the correction of macrocirculatory parameters, is defined as an optimal microcirculatory response in terms of oxygen supply in accordance with the metabolic needs. The loss of coherence between the two haemodynamic systems is a feature of septic shock and is associated with a high risk of morbidity and mortality [3].

In most situations, the restoration of macrohaemo-dynamics should be followed by an improvement in microcirculatory perfusion. However, this is not always the case, particularly in patients with severe septic shock, where targeted therapy to restore microcirculation (e.g. plasma lactate correction) is employed to optimise macrohaemodynamic parameters. This often leads to either over- or underresuscitation, resulting in increased morbidity and mortality rates [4].

In light of these statements, in a comprehensive review of the recent literature, Carlos Sanchez et all. had concluded that the correction of hypotension by early administration of vasopressors in combination with small amounts of intravenous fluid in fluid-responsive patients may prove superior in terms of outcome to the strategy of administering large volumes of fluid prior to the initiation of vasopressors in septic shock [5].

Similarly, coherence between the microcirculation and the interstitial and subcellular (bioenergetic mitochondrial) space is achieved by maintaining an optimal cellular bioenergetic performance and improving organ dysfunction as a result of haemodynamic resuscitation.

Consequently, the concept of coherence (or loss of coherence) between haemodynamic and cellular performers is applicable in both directions: from macro-haemodynamics to mitochondria and vice versa [6].

Although the concept of loss of coherence has been intensively studied in recent decades, it remains poorly understood. This demands systematic research efforts towards early microcirculation resuscitation and its impact on macrohaemodynamics.

Macrocirculatory determinants are represented by cardiac output and systemic arterial pressure. Micro-circulatory determinants are the convective oxygen transport (flow of oxygen-carrying red blood cells (RBC)) and diffusion (the distance oxygen has travelled from RBC to cell mitochondria). Parameters that describe the convective capacity (RBC velocity and distribution) and the diffusive capacity (functional capillary density) are the main evaluation instruments for assessing the functional status of microcirculation. There are numerous methods to evaluate the microcirculation of a patient, but many extremely expensive. The capillary refill time (CRT) is the simplest and most accessible clinical method and can be used as a routine method for critical cases. CRT has proven its effectiveness in the ANDROMEDA-SHOCK study in septic patients, where peripheral perfusion-targeted resuscitation may have resulted in lower mortality and faster resolution of organ dysfunction when compared to a lactate-targeted resuscitation strategy [7]. However, a recent meta-analysis suggested that CRT may not be an accurate predictor of adverse events or death in patients at risk. Nevertheless, the accuracy of CRT can be improved when high-quality CRT measurement is performed [8].

Among the devices used for microcirculation evaluation, methods that use optical properties of haemoglobin and laser-based techniques are of particular interest. The newest generation of hand-held microscopes (HVM) allows proper image acquisition of high-quality videos and detect in the sublingual mucosa (or any other organ with a transparent surface) the parameters that characterize the convective and diffusive determinants of microvascular oxygen transport [9]. However, although HVM has scientific value as a tool in clinical research, it is important to know that it is not ready for routine clinical use. This is due to the increased costs and reduced availability, the need for education and training of the users, the limitation in the accessibility of the place to be investigated, the lack of evidence in terms of benefit in reducing morbidity and mortality [10]. The review of Duranteau et al. highlights evaluation tools and microcirculation analysis in critically ill patients [11].

The structural and functional complexity of the microcirculation can only be understood when considered in the context of its relationship with the interstitium and the cellular space surrounding it. The interconnection between the intravascular environment of the microcirculation and the interstitial space is mediated through the endothelial glycocalyx, which is regarded as a “permeability barrier”. The capillary wall is lined by a single cell layer of endothelial cells, which play a pivotal role in maintaining vascular integrity, homeostasis, vasomotor control and immunological defence. Endothelial cells synthesise a range of molecules with important biological roles and vascular effects. The most representative key mediators of the vascular tone are nitric oxide (NO), prostacyclin (both vasodilators) and endothelin-1 (a potent vasoconstrictor).

The luminal surface of endothelial cells is coated by the glycocalyx, a mesh-like network of proteoglycans and glycoproteins. The endothelial glycocalyx plays a crucial role in maintaining vascular homeostasis, exhibiting anti-adhesive and antithrombotic effects, and protecting the endothelium from oxidative stress. It also regulates the barrier function and the vascular permeability as well as modulating erythrocyte rheology. By binding the serum albumin, it maintains colloid osmotic pressure and contributes to maintaining vascular barrier integrity.

Endothelial cell and glycocalyx destruction (also known as endotheliopathy) occurs in different situations, such as ischemia-reperfusion injury, hypoxia, hyperoxia, severe inflammation, sepsis, haemorrhagic shock, hyperglycaemia and excessive shear stress. The aforementioned events induce severe modifications in the structural architecture of the endothelial glycocalyx, including an increased egress of leukocytes, mechanotransduction impairment, loss of coagulation control and antioxidant defence and increased vascular permeability. Currently, the endothelial glycocalyx is unanimously accepted as a crucial mediator of endothelial homeostasis, heavily involved in the sepsis pathophysiology [12,13].

Another point of interest is the pathway of the oxygen molecule via the cytoplasm from the capillary to the mitochondria, where the process of oxidative phosphorylation takes place. Surprisingly, there is a lack of knowledge regarding this aspect. Experimental studies and computer models show that oxygen diffusion into cells is strongly related to its lipid solubility. This is achieved through a channelling process, especially near the midplane of lipid bilayers. It appears that diffusion via lipid networks is the preferred pathway, while diffusion via interstitial fluid is disadvantageous due to the low solubility of oxygen in aqueous fluid and the limited extracellular space between cells. Diffusion via cytosol is also hampered by the low solubility of oxygen in cytosol and subcellular structures such as membranous organelles [14].

In theory, any alteration to the structural integrity, volume, or composition of cells, including the destruction of cell membranes, the formation of oedema, or any other phenomenon, can additionally impede the rate of oxygen diffusion within the pathway towards the mitochondria.

The data presented demonstrate that microcirculation employs sophisticated physiological mechanisms to facilitate the transfer of nutrients and vital gases (mainly oxygen). Given its complex structure and the significance of the vascular barrier, any impairment of its integrity can result in significant adverse outcomes. In these circumstances, it can reasonably be assumed that these impairments represent a significant issue in the initiation and persistence of the “absence of coherence”.

From a physiological perspective, it is evident that the maintenance of microcirculatory integrity is of paramount importance. Expressed metaphorically, concerning cell populations, the microcirculation can be likened to the Greek mythological titan Atlas, who carried the weight of the world on his shoulders [15]. In considering the potentially devastating consequences of disruptions in microvascular function, it is once again illustrative to return to the Greek mythology in a metaphorical sense. In this context, a condition of dysfunctional microcirculation can quickly devolve into a Sisyphean condition [16].

## References

[1] Weil MH, Shubin H (1967). Diagnosis and Treatment of Shock.

[2] Weil MH, Henning RJ (1979). New concepts in the diagnosis and fluid treatment of circulatory shock. Thirteenth annual Becton, Dickinson and Company Oscar Schwidetsky Memorial Lecture. Anesth Analg.

[3] Ince C (2015). Hemodynamic coherence and the rationale for monitoring the microcirculation. Crit Care.

[4] Bakker J, Ince C (2020). Monitoring coherence between the macro and microcirculation in septic shock. Curr Opin Crit Care.

[5] Carlos Sanchez E, Pinsky MR, Sinha S, Mishra RC, Lopa AJ, Chatterjee R (2023). Fluids and Early Vasopressors in the Management of Septic Shock: Do We Have the Right Answers Yet?. J Crit Care Med..

[6] Boros M, Bauer I (2021). Editorial: Microcirculation Guided/Targeted Resuscitation. Front Med (Lausanne).

[7] Zampieri FG, Damiani LP, Bakker J, Ospina-Tascón GA, Castro R, Cavalcanti AB, Hernandez G (2020). Effects of a Resuscitation Strategy Targeting Peripheral Perfusion Status versus Serum Lactate Levels among Patients with Septic Shock. A Bayesian Reanalysis of the ANDROMEDA-SHOCK Trial. Am J Respir Crit Care Med..

[8] Jacquet-Lagrèze M, Pernollet A, Kattan E, Ait-Oufella H, Chesnel D, Ruste M, Schweizer R, Allaouchiche B, Hernandez G, Fellahi JL (2023). Prognostic value of capillary refill time in adult patients: a systematic review with meta-analysis. Crit Care..

[9] Edul VK, Gutierrez FJ (2023). Devices for assessing microcirculation. Curr Opin Crit Care.

[10] Jacquet-Lagrèze M, Magnin M, Allaouchiche B, Abrard S (2023). Is handheld video microscopy really the future of microcirculation monitoring?. Crit Care..

[11] Duranteau J, De Backer D, Donadello K, Shapiro NI, Hutchings SD, Rovas A, Legrand M, Harrois A, Ince C (2023). The future of intensive care: the study of the microcirculation will help to guide our therapies. Crit Care.

[12] Aldecoa C, Llau JV, Nuvials X, Artigas A (2020). Role of albumin in the preservation of endothelial glycocalyx integrity and the microcirculation: a review. Ann Intensive Care.

[13] Dull RO, Hahn RG (2022). The glycocalyx as a permeability barrier: basic science and clinical evidence. Crit Care.

[14] Pias SC (2021). How does oxygen diffuse from capillaries to tissue mitochondria? Barriers and pathways. J Physiol.

[15] https://www.merriam-webster.com/dictionary/atlas.

[16] https://www.merriam-webster.com/dictionary/Sisyphus.

